# Sensitive imaging of intact microvessels *in vivo* with synchrotron radiation

**DOI:** 10.1107/S2052252520008234

**Published:** 2020-07-11

**Authors:** Feixiang Wang, Panting Zhou, Ke Li, Muyassar Mamtilahun, Yaohui Tang, Guohao Du, Biao Deng, Honglan Xie, Guoyuan Yang, Tiqiao Xiao

**Affiliations:** a Shanghai Synchrotron Radiation Facility/Zhangjiang Laboratory, Shanghai Advanced Research Institute, Chinese Academy of Sciences, Shanghai 201204, People’s Republic of China; b Neuroscience and Neuroengineering Research Center, Med-X Research Institute and School of Biomedical Engineering, Shanghai Jiao Tong University, Shanghai 200030, People’s Republic of China; c Shanghai Institute of Applied Physics, Chinese Academy of Sciences, Shanghai 201800, People’s Republic of China; d University of Chinese Academy of Sciences, Beijing 100049, People’s Republic of China

**Keywords:** microvessels, vascular diseases, move contrast X-ray imaging, *in vivo* imaging, X-ray angiography, contrast agents

## Abstract

Early stages of diseases, including stroke, hypertension, angiogenesis of tumours, spinal cord injuries, *etc*., are closely associated with the lesions of microvasculature. Rodent models of human vascular diseases are extensively used for the preclinical investigation of the disease evolution and therapy with synchrotron radiation. Therefore, non-invasive and *in vivo* X-ray imaging with high sensitivity and clarity is desperately needed to visualize the microvessels in live-animal models. Contrast agent is essential for the *in vivo* X-ray imaging of vessels and angiomatous tissue. Because of the non-rigid motion of adjacent tissues, the short circulation time and the intermittent flow of contrast agents in vessels, it is a great challenge for the traditional X-ray imaging methods to achieve well defined images of microvessels *in vivo*. In this article, move contrast X-ray imaging (MCXI) based on high-brightness synchrotron radiation is developed to overcome the intrinsic defects in conventional methods. Experiments with live rodents demonstrate the practicability of the MCXI method for sensitive and intact imaging of microvessels *in vivo*.

## Abstract 2

In this article, move contrast X-ray imaging (MCXI) based on high-brightness synchrotron radiation is developed. Experiments with live rodents demonstrate the practicability of the MCXI method for sensitive and intact imaging of microvessels *in vivo*.

## 1. Introduction   

Early stages of diseases, including stroke, hypertension, angiogenesis of tumours, spinal cord injuries, *etc*., are closely associated with the lesions of microvasculature (Ayala-Domínguez & Brandan, 2018 ▸; Cao *et al.*, 2019 ▸; Gu *et al.*, 2019 ▸; Guan *et al.*, 2012 ▸; Kidoguchi *et al.*, 2006 ▸; Kiessling *et al.*, 2004 ▸; Li *et al.*, 2019 ▸; Lin *et al.*, 2015 ▸; Liu *et al.*, 2010 ▸; Tokunaga *et al.*, 2018 ▸; Umetani *et al.*, 2007 ▸; Wang *et al.*, 2017 ▸, 2019 ▸). Rodent models of human vascular diseases are extensively used for the preclinical investigation of the disease evolution and therapy. Therefore, non-invasive and *in vivo* X-ray imaging with high clarity is desperately needed to reveal microvessels in live-animal models (Starosolski *et al.*, 2015 ▸). Taking advantage of their high flux density, synchrotron radiation X-rays are currently used for the high spatial and temporal resolution imaging of microvessels of rodent models *in vivo*. In addition, contrast agent is essential for the *in vivo* imaging of vessels and angiomatous tissue (Layer, 2019 ▸). Conventional iodine-based contrast agents have been extensively used to enhance the contrast between vessels against their complicated surroundings. Current agents for X-ray angiography have the following limitations: short circulation time, occasional renal toxicity and poor contrast in large patients (Hainfeld *et al.*, 2006 ▸). Nanoparticles are employed to overcome the shortcomings of conventional agents (Hainfeld *et al.*, 2006 ▸; Han *et al.*, 2019 ▸; Hyafil *et al.*, 2007 ▸; Park *et al.*, 2017 ▸). However, before the use of nanomaterials for *in vivo* disease diagnosis and treatment, plenty of preclinical investigation is required (Park *et al.*, 2017 ▸).

Temporal subtraction X-ray imaging combined with contrast agents is currently used to eliminate the interference of other tissues. Because of the uncontrollable movement of tissues, short circulation time and intermittent flow of contrast agents in vessels during *in vivo* imaging, traditional digital subtraction X-ray imaging does not work well in obtaining sensitive and intact imaging of microvessels in animal models. Non-intrusive and precise imaging for tumour angiogenesis is critical in the accurate assessment of cancer diagnosis and prognosis. X-ray microtomography is now the dominant method for the studies of angiogenesis *in vitro*, but X-ray imaging modalities applicable to the *in vivo* visualization of the microvessels are quite limited (Ayala-Domínguez & Brandan, 2018 ▸; Gu *et al.*, 2019 ▸; Kiessling *et al.*, 2004 ▸; Li *et al.*, 2019 ▸; Liu *et al.*, 2010 ▸). Stroke and hypertension are closely related to the subtle change of microvessels but current X-ray imaging methods still suffer from insufficient imaging resolution for the dynamical and direct observation of rodent cerebrovascular alterations *in vivo* (Guan *et al.*, 2012 ▸; Kidoguchi *et al.*, 2006 ▸; Lin *et al.*, 2015 ▸; Umetani *et al.*, 2007 ▸; Wang *et al.*, 2017 ▸, 2019 ▸; Yuan *et al.*, 2013 ▸). Nanocomposite containing high-*Z* metal elements is used for imaging-guided drug-delivery research in rodent models, and sensitive tracking of these composites *in vivo* will benefit the related diagnosis and therapy (Gradl *et al.*, 2019 ▸; Shi *et al.*, 2014 ▸; Tian *et al.*, 2015 ▸). A hybrid bis­muth/carbon-nanotube contrast agent was successfully used to locate the labelled stem cells with X-ray imaging but *in vivo* studies with animal models are still required to evaluate the efficacy of this material (Hernández-Rivera *et al.*, 2019 ▸). Furthermore, *in vivo* imaging of the rodent spinal cord microvasculature is a challenging task at present because of the significant movement artefacts from the adjacent lungs and heart (Cao *et al.*, 2019 ▸; Tokunaga *et al.*, 2018 ▸). A long circulating blood-pool contrast agent has been adopted for the *in vivo* microtomography of mouse cerebrovasculature and small vessels have been successfully delineated. However, a large dose of contrast agent is required for this method and motion artefacts cannot be eliminated (Layer, 2019 ▸). Therefore, an *in vivo* X-ray imaging method was solicitously expected to delineate microvessels with high sensitivity.

The advantage of X-rays is their high penetration of thick samples for non-destructive imaging of the internal structures, while the short wavelength of X-rays also enables their high spatial resolution. On the other hand, this is also a disadvantage of X-ray imaging. For thick samples, the stack of layered microstructure will deteriorate the spatial resolution. For static structures, X-ray microtomography could solve this problem perfectly. However, this is not the case for *in situ* or *in vivo* imaging of a complex system, which is defined as systems with intricate structure, complex components and complicated relative movement. Obviously, a live animal is a typical example of a complex system. This implies a great challenge for conventional methods to delineate the complicated microvessels in a live rodent. A time-domain filtering method was employed to eliminate the motion artefacts in digital subtraction angiography (Bray *et al.*, 1985 ▸; Nelson *et al.*, 1982 ▸). However, further substantive improvements are still awaited. In this article, a move contrast X-ray imaging (MCXI) method based on synchrotron radiation is developed to solve the above-mentioned problems for sensitive and intact imaging of rodent microvessels *in vivo*.

## 2. Method   

As is well known, though most organs or tissues inside the body are moving all the time, they should move in different frequencies. By analyzing the frequency of intensity variation at each pixel of the image frame, it should be possible to differentiate the migration of contrast agent along vessels from the movement of other tissues and then image them independently. Based on this idea, MCXI is proposed for sensitive and intact imaging of microvessels *in vivo*, in which the imaging contrast stems from the moving characteristics of interested objects. To record the moving process of all the tissues, a video for the perfusion process of contrast agent in a live animal is recorded frame by frame in a time sequence (see Videos S1–S4 in the Supporting information). This video actually acts as primary data for MCXI reconstruction in which the grayscale variation resulted from the movement of contrast agent and tissues is defined as move contrast. In principle, the intensity signal *I*(*x*, *y*, *t*) at each pixel of the frames captured by an X-ray CCD camera can be expanded to a series of sinusoidal signals in the frequency domain (Wang, 2019 ▸).
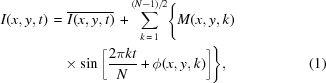
where *N* is the total number of sequenced images and is assumed to be odd. 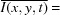 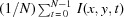 is the average intensity during a period time lasting through all of the data-acquisition process. The term 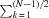 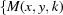 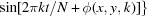 represents the intensity-fluctuation components resulted from the contrast-agent-enhanced blood flow or moving of organs and tissues. *M*(*x*, *y*, *k*) and ϕ(*x*, *y*, *k*) are the amplitude and phase of these components at the corresponding frequencies, respectively. Then the signal obtained from blood flow or the moving of organs and tissues can be demodulated independently by applying band-pass filters in the frequency domain, which can be expressed as a series of AC signals,
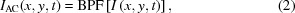
where BPF[] denotes the band-pass filtering to the time evolution of intensity recorded by the X-ray detector. Raw temporal signals of arteries, veins and the background tissue are transferred from the time domain to the frequency domain, the significant differences between the vascular region and the moving tissues can be distinguished in the frequency spectrum.

**Figure 1:**
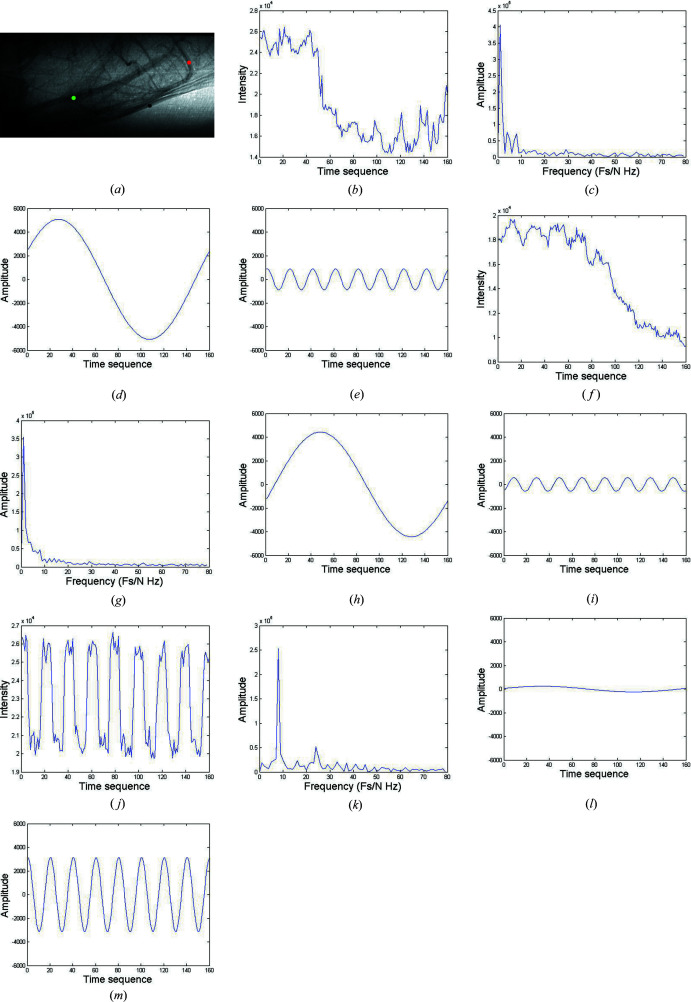
Data processing for MCXI-based angiography, including one of the original projections for angiography (*a*); the time-sequenced signal at an artery (*b*), a vein (*f*) and moving tissue (*j*), extracted from the video of the time-sequenced projections (see Video S3); the time-domain frequency spectra of an artery (*c*), a vein (*g*) and moving tissue (*k*); the band-filtered spectra at 1*F*_s_/*N* Hz for an artery (*d*), a vein (*h*) and moving tissue (*i*); and the band-filtered spectra at 8*F*_s_/*N* Hz for an artery (*e*), a vein (*i*) and moving tissue (*m*).

To illustrate the procedure for data processing in MCXI-based angiography, an example is given as follows. One of the original projections for angiography (see Video S3) is shown in Fig. 1 ▸(*a*). The locations marked by the red (artery) and green (vein) dots refer to blood vessels and the location marked by the black dot is the background tissue. The infusion process can be observed apparently at the location of the vessel in Video S3. The background tissue marked by the black dot moves constantly at a wide-range frequency owing to breath and convulsion of the mouse, which is usually expected to be stationary in traditional temporal subtraction angiography. The intensity variation with time at these three points (red, green and black dots) is extracted from the video and shown in Figs. 1 ▸(*b*), 1 ▸(*f*) and 1 ▸(*j*), respectively. It is obvious that the curves for the vessels are similar to a step signal and that the curve for the background tissue appears periodic.

The raw temporal signals of the artery, the vein and the background tissue are transferred from the time domain to the frequency domain by fast Fourier transform. As shown in Figs. 1 ▸(*b*), 1 ▸(*f*) and 1 ▸(*j*), significant differences between the vascular region and the moving tissue can be observed in the frequency spectrum. In this case, *F*_s_ denotes the sampling frequency for the image sequence and *N* denotes the number of images collected for angiography. According to the band-filtering spectrum shown in Figs. 1 ▸(*c*), 1 ▸(*g*), 1 ▸(*k*), 1 ▸(*d*), 1 ▸(*h*) and 1 ▸(*l*), it is obvious that the wave energy at the vascular region mainly concentrates at a frequency of *F*_s_/*N* Hz and that the wave energy for the moving tissue focuses at a frequency of 8*F*_s_/*N* Hz. With band filtering in the frequency domain for each pixel, images corresponding to different frequencies can be reconstructed independently.

In this way, the track of contrast agent or the movement of organs inside the body can be imaged independently and the mutual interference is eliminated. As a result, the sensitivity of the MCXI and the signal-to-noise ratio of the vessel images can be greatly improved. The high sensitivity of the MCXI method implies that imaging of microvessels can be performed with a lower-luminance X-ray source or with a lower dose of contrast agent. In MCXI, the entire process of blood perfusion can be reconstructed from the time-sequenced projections whether the contrast agent is a constant or intermittent perfusion in the vessels. For arterial and venous vessels, the dynamic signal [shown in Figs. 1 ▸(*d*) and 1 ▸(*h*)] not only contains the amplitude information that represents the concentration of contrast agent, but also contains the phase information that represents the perfusion moment of the contrast agent. By differentiating the time when the contrast agent arrives at the blood vessels, arteries and veins can be discriminated and the overlapping effect of these two sets of vessel systems can be eliminated.

## 3. Experimental results   

### 3.1. Experimental setup   

**Figure 2:**
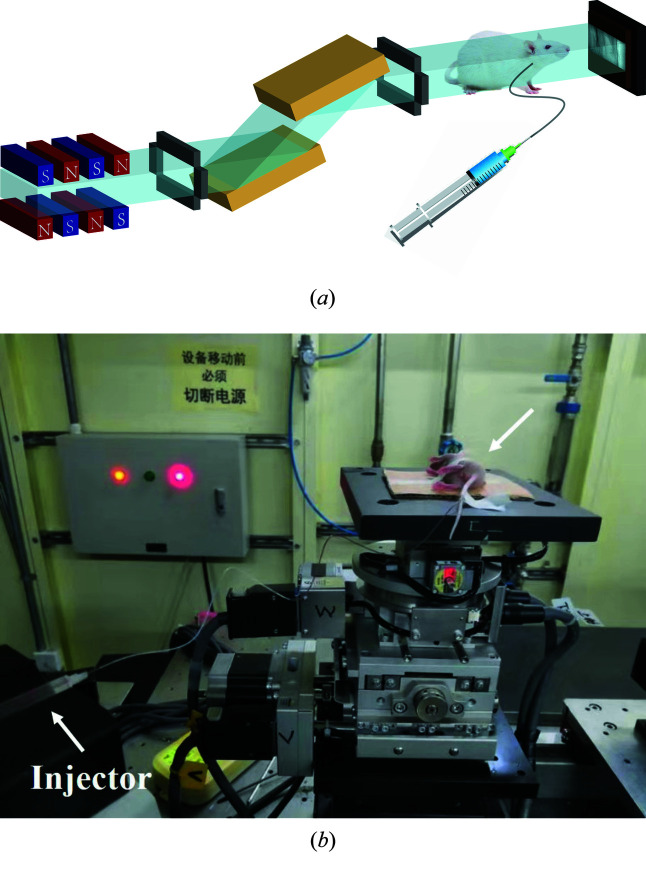
The experimental setup for MCXI with synchrotron X-rays, where (*a*) is the optical layout and (*b*) is a picture of the sample stage.

The experiments were carried out at the BL13W beamline of the Shanghai Synchrotron Radiation Facility (SSRF), as shown in Fig. 2 ▸. After a double-crystal monochromator downstream of a wiggler source, X-ray energy for the monochromatic beam is set to 33.3 keV, *i.e.* the *K* edge of iodine, to achieve significant contrast enhancement. The output flux for the experiments was 2.38 × 10^10^ photons s^−1^ mm^−2^. Adult-male ICR mice weighing 25 to 30 g were anaesthetized with ketamine (100 mg kg^−1^) and xylazine (10 mg kg^−1^, Sigma, San Louis, Missouri) intraperitoneally. Non-ionic iodine (Ipamiro, Shanghai, China) with a concentration of 280 mg I ml^−1^ (primary 350 mg I ml^−1^ diluted to 80% volume ratio with saline) was selected as the contrast agent. An angiographic tube was inversely inserted along the external carotid artery into the bifurcation of common carotid artery before contrast injection. During image taking for the MCXI, a total volume of 180 µl iodine was injected through external carotid arteries into internal carotid arteries at an injecting rate of 133.3 µl s^−1^, which was controlled by a micro-syringe pump (LSP01-1A, Longerpump, Baoding, China). A PCO X-ray CCD camera (a pixel size of 6.5 × 6.5 µm, a field of view of 13 × 13 mm, PCO-TECH Inc., Germany) was selected as the detector and placed 65 cm away from the sample. The image sequence was taken at a frame rate of 100 frames s^−1^ with an exposure time of 10 ms for a single projection.

### 3.2. Demonstration with live rodents   

**Figure 3:**
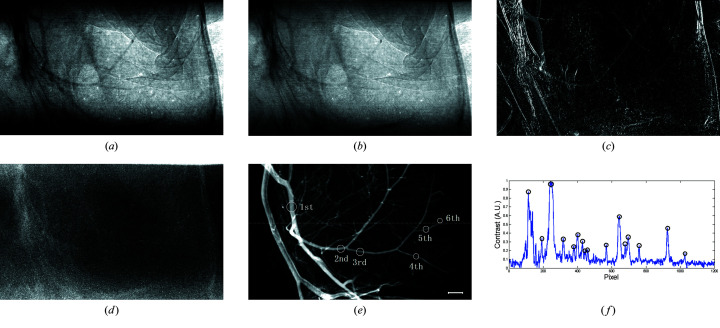
The experimental results of MCXI with synchrotron X-rays, where (*a*) represents the single image frames of the raw angiogram, (*b*) is the time-averaged image of the raw angiogram, (*c*) is the image of moving tissues reconstructed by MCXI, (*d*) represents the high-frequency noises reconstructed by MCXI, (*e*) represents the intact blood vessels delineated by MCXI and (*f*) is the intensity profile along the horizontal dashed line in Fig. 3 ▸(*e*). The scale bar represents 500 µm.

Live mice were used as animal models to verify the proposed MCXI because the diameter of blood vessels of mice is much smaller than that of humans. Protocols of animal experiments used in this study were approved by the Institutional Animal Care and Use Committee (IACUC), Shanghai Jiao Tong University, Shanghai, China. High-brightness synchrotron radiation X-rays were used as the light source to achieve high spatial resolution (Ayala-Domínguez & Brandan, 2018 ▸; Hyafil *et al.*, 2007 ▸). Middle cerebral artery territory, which is generally associated with middle cerebral aneurysm and middle cerebral artery occlusion, is selected as the interested vasculature for the experiments. In the single image frame of the raw angiography data, as shown in Fig. 3 ▸(*a*), the image of blood vessels is seriously degraded by the superposition of bones and tissues, even though contrast agent is injected to enhance the vasculature. The intact and coherent images of the blood vessels can hardly be made out. Moreover, the blood vessels were significantly displaced during the infusion process of contrast agent accompanied by the breath and tremor of the mouse. In traditional temporal subtraction angiography, the movement of other tissues is usually inevitable for a live body and a simple subtraction to a so-called static background is hard to achieve for high-contrast and high-sensitivity imaging of vasculature.

According to the MCXI method introduced above, blood flow and the moving tissues in the primary images can be distinguished according to their moving frequencies in the time domain. The results obtained by MCXI are shown in Fig. 3 ▸, in which the averaged background image [similar to the mask image of the angiogram, Fig. 3 ▸(*b*)], tissues moving in lower frequency [Fig. 3 ▸(*c*)], high-frequency noises [Fig. 3 ▸(*d*)] and the blood vessels in the fundamental frequency [Fig. 3 ▸(*e*)], are given independently. Fig. 3 ▸(*e*) shows the mouse middle cerebral artery, in which an intact microvascular network is achieved with high clarity and high spatial resolution, and the noises and the structure of other tissues are clearly removed. In MCXI, the averaged background image usually represents the zero-frequency component, while the blood flow enhanced by the contrast agent is the fundamental frequency, the breath and tremor-induced movement has a higher frequency, and the noises from other sources locate at the high-frequency band. In this way, the trace of blood flow, *i.e.* the blood vessels, can be explicitly delineated from the time-sequenced projection images without the interference of other tissues and noises. In addition, higher-frequency motion of tissues, such as the movement induced by breath and tremor [Fig. 3 ▸(*c*)], can be reconstructed independently, which may provide additional information for the blood-vessel analysis. The stripping of the high-frequency noises [Fig. 3 ▸(*d*)] has greatly improved the contrast-to-noise ratio of the blood-vessel image [Fig. 3 ▸(*e*)]. Fig. 3 ▸(*d*) shows a distribution of high-frequency noises obtained from the movement of the blood vessels and the adjacent tissues. In this figure, the shadow of the blood vessels can still be determined which means that the vessel itself also has high-frequency movement, possibly caused by a heartbeat. Fig. 3 ▸(*b*) shows the averaged image in zero frequency of the interested area, which resembles the mask image in traditional temporal subtraction X-ray angiography.

To show the advantages of MCXI in high contrast-to-noise ratio and high spatial resolution, a detailed demonstration is given in the following. Intensity profiles and vessel diameter are employed to quantitatively evaluate the image quality of the proposed method. In Fig. 3 ▸(*e*), the main microvessels up to sixth branches indicated by the circles can be explicitly revealed by MCXI. Intensity profiles along the dashed line in Fig. 3 ▸(*e*), in which a line section was selected, were used to analyze the ability of the MCXI method in identifying microvessels. A black circle is used to note a resolved vessel in the profiles. Fifteen vessels are resolved by MCXI, as shown in Fig. 3 ▸(*f*), according to the intensity profile. By checking the full width at half-maximum (FWHM) value of picks in the profile, the finest vessel resolved by MCXI is estimated to be 18 µm. As a full-field imaging method, MCXI exhibits its ability to image an intact vessel system including main vessels and abundant microvessels simultaneously, which will be advantageous for diagnosis and therapy of vascular diseases. From the experimental results mentioned above, MCXI can extract blood microvessels from the complex background of other tissues. The effect of factors, including the mask image of the interested area, movement of other tissues and the high-frequency noises, on the image quality of microvessels can be almost completely removed. As a result, the sensitivity and spatial resolution of X-ray imaging of microvessels *in vivo* can be greatly improved.

### 3.3. Imaging with a low dose of contrast agent   

**Figure 4:**
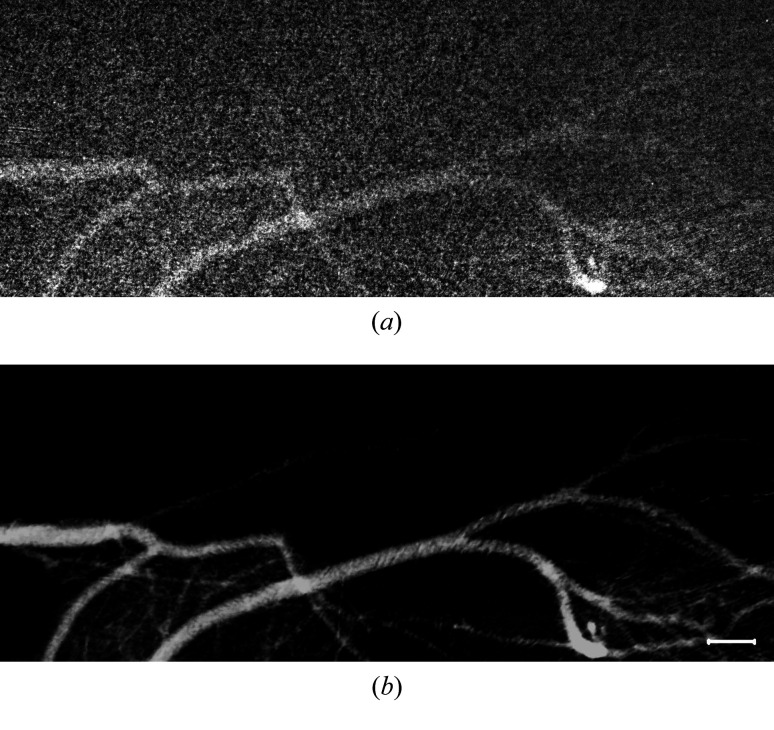
Delineating a mouse middle cerebral artery with a low-dose contrast agent, where (*a*) represents traditional temporal subtraction X-ray angiography and (*b*) represents MCXI, with the contrast agent diluted to 10% of normal concentration. The scale bar represents 500 µm.

The dosage of hypertonic iodide contrast agent is closely related to the occurrence of contrast-induced nephropathy (CIN). Correlation analysis has shown that the nephrotoxicity of contrast agent is positively correlated with the dose of contrast agent. For example, the incidence of CIN decreased by 15% when the amount of contrast agent was reduced by 45 ml (Mamoulakis *et al.*, 2017 ▸; Rear *et al.*, 2016 ▸). At present, the main solution to prevent CIN is to reduce the dosage of contrast agent. As demonstrated above, the high sensitivity of the proposed MCXI method enables the possible use of a much lower concentration of contrast agent for the *in vivo* imaging of microvessels and so the agent dose can be significantly reduced. Experiments on rodent models were carried out to evaluate the sensitivity of MCXI to contrast agent. In the experiments, a 10% concentration of standard contrast agent was injected into the external carotid artery of the mouse, with the same volume (180 µl) and the same experimental conditions as that shown in Fig. 3 ▸. The mouse middle cerebral artery territory was selected as the object of interest. As shown in Fig. 4 ▸(*b*), the vasculature of the mouse brain can be clearly revealed by MCXI even though the normal contrast agent is diluted to 10%. For comparison, the angiogram by traditional temporal subtraction is also given in Fig. 4 ▸(*a*). It is obvious that owing to the sharp decrease in the concentration of contrast agent, the traditional temporal subtraction X-ray angiography is no longer effective in delineating cerebral vessels. Compared with Fig. 4 ▸(*a*), the cerebrovascular in Fig. 4 ▸(*b*) gives more details of small blood vessels with a higher contrast-to-noise ratio. These experimental results demonstrate that MCXI can improve the sensitivity of X-ray imaging of microvessels *in vivo* by one order of magnitude. This means that MCXI combined with a low concentration of contrast agent may provide a new strategy of angiography for patients with renal insufficiency to reduce the incidence of CIN.

### 3.4. Imaging with low-flux X-rays   

**Figure 5:**
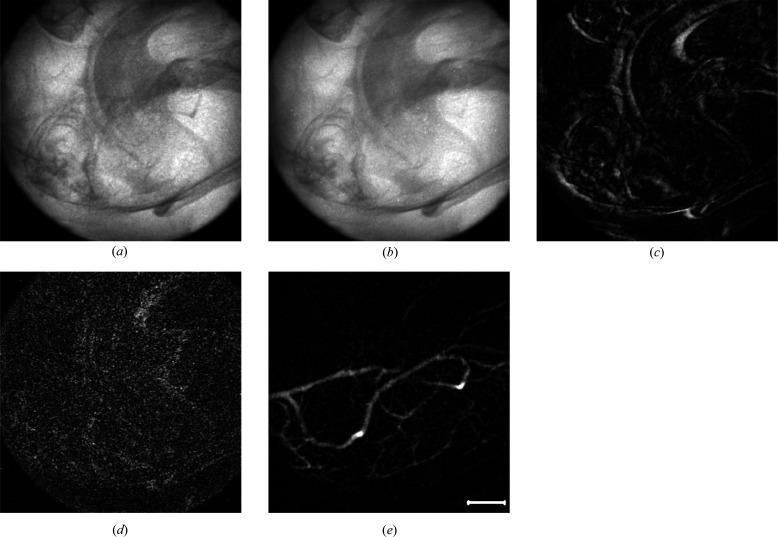
The experimental results for MCXI with a X-ray tube, where (*a*) is a single image frame of the raw angiogram, (*b*) is the time-averaged image of the raw angiogram; (*c*) is the image of moving tissues reconstructed by MCXI; (*d*) represents the high-frequency noises reconstructed by MCXI and (*e*) represents the blood vessels delineated by MCXI. The scale bar represents 2000 µm.

As mentioned above, high-brightness synchrotron X-rays from a wiggler source at SSRF were used to take the sequenced images with a high frame rate and short exposure time. If the MCXI is sensitive enough to enable the use of a conventional laboratory X-ray source, its applications for preclinical research on vessel diseases will be promoted more intensively. Therefore, further experiments were carried out to verify the practicability of the MCXI method with a X-ray tube as the light source. In the experiments, the micro-focus X-ray tube with tungsten target (Hamamatsu, L8121-03, 75 W) at 80 kV and 500 µA was employed. The focal spot size of the X-ray beam is 50 µm and the divergence angle is 43°. The same X-ray CCD detector was employed for the experiments but operated at four binning mode to improve the detecting sensitivity, *i.e.* with an effective pixel size of 26 µm. The CCD camera was placed 10 cm away from the sample stage and used to acquire the time-sequenced images at a frame rate of 5 frames s^−1^ and an exposure time of 200 ms. The difficulties for this experiment are obvious: low frame rate and relatively long exposure time may not keep step with the breath and tremor of the live mouse, and the interference from moving tissues may not be eliminated. All the animal surgical procedures and experimental preparation for the angiography are the same as the experiments carried out with the synchrotron X-ray source. Similarly, the middle cerebral artery is again selected as the object of interest for the experiments. The video of raw data is shown in Video S2. From the single image frame of the raw angiograms shown in Fig. 5 ▸(*a*), the microvessels cannot be distinguished at all and it is also difficult to discern the main vessels in the complex background. This means that traditional temporal subtraction X-ray angiography is ineffectual in this case. Furthermore, because the brightness of the X-ray tube is far inferior to that of synchrotron radiation, the images shown of the raw data are quite noisy. The intensity fluctuation in the image field caused by noises will dramatically deteriorate the image quality of vessels. Therefore, image pre-processing using median filtering is implemented for each frame of the raw data before the image reconstruction by MCXI.

The experimental results of MCXI with the X-ray tube are shown in Fig. 5 ▸. In Fig. 5 ▸(*b*), the averaged image in zero frequency is reconstructed separately. The complex tissue motion in high frequency is shown in Fig. 5 ▸(*c*) and the higher-frequency noise is shown in Fig. 5 ▸(*d*). In Fig. 5 ▸(*e*), the main vessels and microvessels in fundamental frequency are revealed with acceptable clarity. Results in Figs. 5 ▸(*c*) and 5 ▸(*d*) show that the low frame rate and long exposure time substantially amplify the noises. It is hard to observe the blood vessel directly from the projection image at the lower left quadrant of Fig. 5 ▸(*a*) because of the severe interference of tissue movements at the same location. However, the image in Fig. 5 ▸(*e*) indicates that intact blood vessels can still be delineated by MCXI, though apparent noises are also introduced because of the insufficient flux density of X-ray tubes. As described in the method for data processing, the MCXI method analyzes the time-varying signal at each pixel separately in the time-domain frequency space, and the signal of contrast media flow can be clearly distinguished from the signals resulted by moving tissues and high-frequency noises according to their frequency differences. With the interference of moving tissues and noises eliminated, the ability of MCXI in extracting weak signals is improved significantly. This experiment demonstrates that the proposed MCXI is also applicable to imaging vessels *in vivo* with an X-ray tube. However, limited by the much lower flux density of X-ray tubes, the achieved spatial resolution and signal-to-noise ratio of the vessel image is not comparable with that obtained with synchrotron sources, which confirms the advantage of synchrotron radiation for microvessel imaging *in vivo*.

### 3.5. Delineating the intact vasculature   

The results given above are all based on the amplitude information of the move contrast. From the series expansion of image intensity in MCXI, the additional phase information which denotes the chronological sequence of the moving process, such as contrast-agent perfusion, is still not used. Further experiments were carried out to demonstrate the outreaches of MCXI in reconstructing the whole trajectory along which the contrast agent passes through. In this way, the intact vasculature can be delineated.

**Figure 6:**
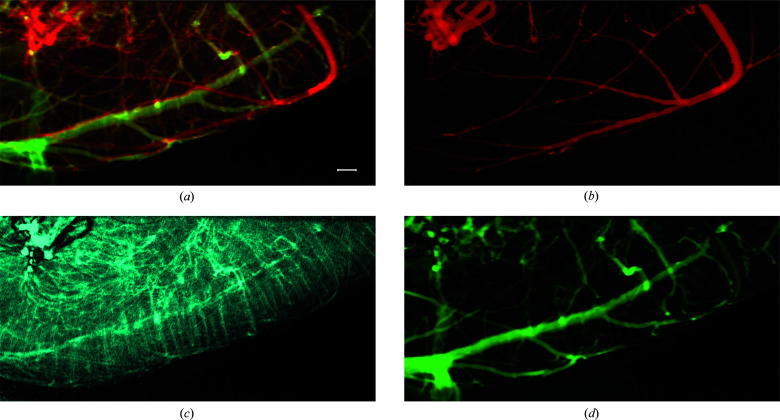
Intact vasculature imaging with MCXI, including the whole vasculature (*a*), arteries (*b*), a capillary image with insufficient resolution (*c*) and veins (*d*). The scale bar represents 500 µm.

Based on the difference of time when the front end of the contrast agent appears in the vessels (Video S3), the perfusion process of contrast agent in rodent middle cerebral vessels was recorded and then used to reconstruct the vasculature in mouse brain. All the experimental conditions were consistent with those used for Fig. 3 ▸. At the vascular region, the dynamic signal *I*_AC_(*x*, *y*, *t*) not only contains the amplitude information which represents the concentration of contrast agent, but also the phase information which denotes the moment of the contrast-agent perfusion. Phase values reflect the perfusion process of contrast agent in the blood vessels and can be used to determine the time. As is well known, the contrast agent passes through the arteries, the capillaries network and finally the veins. Based on the phase value of the dynamic signal *I*_AC_(*x*, *y*, *t*), the whole trajectory of contrast agent in vessels can be reconstructed. When the trajectory of contrast agent, *e.g.* blood vessels, is coloured according to the phase values of the dynamic signal *I*_AC_(*x*, *y*, *t*), the intact vasculature in the interested area can be exhibited intuitively. As shown in Fig. 6 ▸(*a*), the chronological sequence for agent perfusion is presented according to a colour bar from red to green. However, showing the whole circulation process in a single image will inevitably lead to overlapping of the arteries, capillaries and veins. Furthermore, the veins enhancement may obscure the visualization of arteries and lead to a incorrect diagnosis. The ability to separately image arteries and veins is usually a challenge for temporal subtraction angiography methods. After the contrast agent enters the vein, there is still agent flowing along the artery, which makes it difficult for temporal subtraction angiography to obtain a pure venous image. However, MCXI can solve this problem with ease and image the arteries and veins independently according to the chronological sequence with contrast agent perfused through the vessels. The perfusion process is shown in Video S3, image frames of which were employed to reconstruct the vessel images. For example, the video frames of venous perfusion can be intercepted and used to reconstruct a pure venous image. At this time, although the arteries still had contrast agent flowing through them, their motion characteristics were different from those of the veins. Separately reconstructed images of the arteries, capillaries and veins are shown in Figs. 6 ▸(*b*), 6 ▸(*c*) and 6 ▸(*d*), respectively. In Fig. 6 ▸(*b*), the arteries are relatively simple and the vessels look straight with fewer branches. Limited by spatial resolution in the experiments, the capillaries are not resolved separately from the image shown in Fig. 6 ▸(*c*). Moreover, the vasculature of veins in Fig. 6 ▸(*d*) looks more complex than that of the arteries shown in Fig. 6 ▸(*b*). The veins are thicker and more twisted. If lesions of vasculature occur in certain kinds of vessels, the advantage of independent imaging of different vessels with MCXI will greatly benefit the sensitive diagnosis *in vivo*. The ability of MCXI to reconstruct the complete perfusion process of contrast agent may be employed for the investigation of hemodynamics in certain segments of vessels.

### 3.6. Intact imaging of vessels with flashed flow   

In X-ray angiography, only a portion of the vessels are filled with the contrast agent most of the time. The complete perfusion of contrast agent in the vessels is often accidental, and in the ureter and some pathological blood vessels the contrast agent is usually flashed like a pulse. The intermittent recording of contrast agent inside the vessels leads to an incomplete structure of the vessels. Generally, multiple subtraction images at different moments are required to obtain a complete angiogram through image fusion. However, the non-rigid motion of each component in living organisms not only makes the registration algorithm before subtraction useless but it also makes the image fusion of the vessels difficult. In principle MCXI should have the ability to image vessels with flashed flow, combining with contrast agent. The ureter, which is usually a challenge to temporal subtraction imaging methods because of the pulsive flow of contrast agent in the vessels, is selected to further verify the practicability of MCXI.

**Figure 7:**
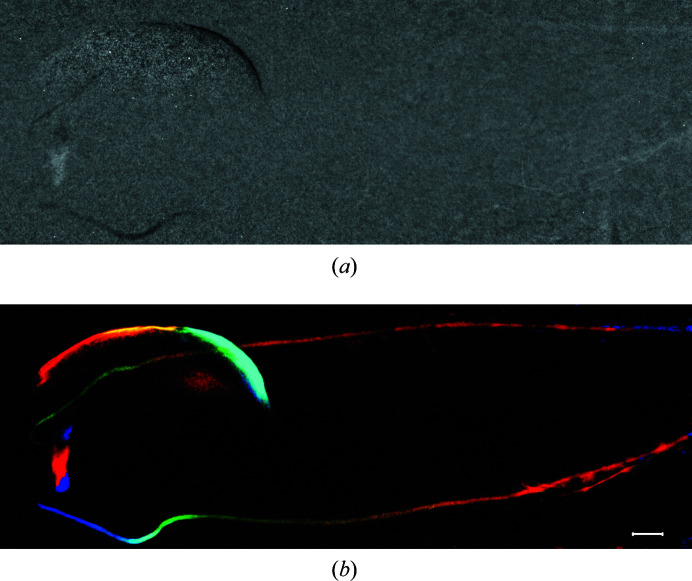
The experiments on a mouse ureter, where (*a*) is the image by temporal subtraction and (*b*) is the image by MCXI with the intact ureter delineated. The scale bar represents 500 µm.

In the experiments, 500 µl (280 mg I ml^−1^) of contrast agent was injected into the tail veins of live mice. Iodide ions were excreted by the kidney and then collected by the ureter and delivered to the bladder. As shown in the video of original data (Video S4), the excretion of the agent is a pulsing process rather than a continuous flow. Fig. 7 ▸(*a*) is the image obtained by traditional temporal subtraction, in which the ureter can hardly be made out. According to the sequenced image frames, the total length of the ureter is ∼12 mm in the imaging area. The length of this intermittent urine bunch containing contrast agent in the ureter is ∼2.2–4.3 mm. The time required for the urinal bunch to pass through the ureter is ∼5 s and the time interval between the two urine clusters is 4.7 s. This means that only one bunch can be captured during the acquisition of image frames. As shown in Fig. 7 ▸(*b*), it is obvious that the intact ureter is delineated almost perfectly by MCXI. Because of the limit of the field of view, the vessels near the kidneys are not imaged in this experiment. A partial contour of the bladder is also observed in Fig. 7 ▸(*b*), which means that a simultaneous shrinkage or inflation of the bladder occurs during the flashed flow of the urinal bunch. This experiment demonstrated that MCXI has the potential for *in vivo* imaging of vessels with flashed flow.

## 4. Conclusions   

Because of the non-rigid motion of adjacent tissues, the short circulation time and the intermittent flow of contrast agents in vessels, it is a great challenge for the traditional X-ray imaging methods to achieve well defined images of microvessels *in vivo*. In this article, MCXI is proposed to overcome the intrinsic defects in conventional methods. The experimental results on live rodents demonstrated that the MCXI method can image the static background, the movement of adjacent tissues, the high-frequency noises and the blood microvessels independently. As a result, the effect of the complex background and uncontrollable movement of organs and tissues on the image quality of microvessels can be eliminated, which means that the sensitivity of *in vivo* X-ray imaging of microvessels can be greatly improved. Diluted contrast agent is used to verify the sensitivity of MCXI to the dose of contrast agent. The experiments on live mice verified that the middle cerebral vascular can be explicitly delineated even though the concentration of contrast agent is diluted to 10% of the normal value. This low-dose characteristic of MCXI will benefit researches which have critical limits on the quantity of contrast agent. Moreover, this method has the potential for diagnosis of patients with diabetes or renal insufficiency (Jepson *et al.*, 2018 ▸). Sensitivity to photons determines whether the MCXI method has the potential for imaging with laboratory X-ray sources rather than a high-brightness synchrotron radiation facility. Experiments carried out with an X-ray tube verified the capability of MCXI for *in vivo* imaging of microvessels in rodent models. This means that the MCXI method developed at synchrotron radiation facilities may be transferred to laboratory researching on vessel diseases with live-animal models, though the spatial resolution is relatively lower than that with synchrotron X-rays.

Moreover, taking advantage of the phase information in MCXI, the whole moving trajectory of contrast agent in vessels, *e.g.* the complete lumen of the vessels, can be delineated. Experiments on rodent models demonstrated that the complete middle cerebral vasculature was successfully reconstructed by MCXI according to the time when the front end of the agent flow arrives at a certain point in the vessels. Accordingly, the whole process of the agent perfusion along the whole blood-vessel system was reconstructed. In this way, arteries and veins or any vessels of interest can be imaged independently, which may open a new way for the research of hemodynamics and the diagnosis of vasculopathy. As a typical example of the intermittent transportation of contrast agent in vessels, the flashed flow of the urinal bunch in the urine-collecting vessels was taken to reconstruct the whole ureter. The experimental results with a live rodent showed that the intact ureter was successfully imaged while the traditional temporal subtraction method was useless. This confirms the ability of MCXI for intact imaging of microvessels with intermittent flow. In conclusion, the proposed MCXI is practicable for sensitive and intact imaging of microvessels *in vivo*, which will provide a novel tool for the preclinical *in vivo* researches on vessel-related diseases and the evaluation of new contrast agents.

## Supplementary Material

Video S1. DOI: 10.1107/S2052252520008234/hf5942sup1.avi

Video S2. DOI: 10.1107/S2052252520008234/hf5942sup2.avi

Video S3. DOI: 10.1107/S2052252520008234/hf5942sup3.avi

Video S4. DOI: 10.1107/S2052252520008234/hf5942sup4.avi
